# Evaluation of screening criteria for palliative care in an emergency department ICU

**DOI:** 10.1186/cc14650

**Published:** 2015-03-16

**Authors:** S Ribeiro, R Carvalho, P Ayres, D Barros, D Roger

**Affiliations:** 1 Hospital das Clinicas School of Medicine, University of São Paulo, Brazil

## Introduction

A high percentage of patients admitted to ICUs fulfill one or more criteria for palliative care. There are currently few comprehensive studies in critical care settings that have set out to examine the association of palliative care screening criteria with adverse patient outcomes.

## Methods

We performed an observational unicentric study on a 12-bed, medical emergency department intensive care unit (EDICU). A three-item palliative care screen was developed from consensus reports. A senior critical care physician screened patients upon admission using these questions during a 10-week period. The questions were: does this patient suffer from a life-limiting disease (end-stage lung, liver, heart or kidney disease, severe neurological disability, extreme frailty, locally advanced or metastatic cancer, advanced-stage AIDS). If the answer to the first question is yes, we proceed to the next one: do you believe this patient will survive to hospital discharge? Answers to those questions were recorded, SAPS III was calculated and all patients were followed until death, discharge or transfer to another center. Differences in mortality and SAPS III score between groups were examined using a Student's *t *test. Proportions were compared using chi-square test. *P *< 0.05 was considered statistically significant.

## Results

During the period, 191 patients were admitted to the EDICU, from which 151 had complete data and follow-up. A total of 63 patients (41.7%) suffered from a life-limiting disease and were evaluated as having a high probability of death in 1 year. This group was further divided between 35 patients who in the moment of initial screening were expected to die in this hospital admission and 28 patients who were believed to survive to discharge. Comparison between these two groups showed patients believed to die at this hospital admission had higher SAPS III scores (66.9 vs. 59, *P *= 0.010) and hospital mortality (48.6% vs. 10.7%, *P *= 0.001).

## Conclusion

A high percentage of patients admitted to our EDICU have life-limiting disease and might benefit from palliative care. These patients can be identified using simple screening questions at admission and positive answers to those questions can be associated with worse outcomes.
